# Characteristics of non-suicidal self-injury and its associations with gender minority stressors among Hungarian transgender and gender diverse adults

**DOI:** 10.1186/s12888-025-06738-y

**Published:** 2025-04-10

**Authors:** Banu C. Ünsal, Zsolt Demetrovics, Melinda Reinhardt

**Affiliations:** 1 Doctoral School of Psychology, ELTE Eötvös Loránd University, Budapest, Hungary; 2 Institute of Psychology, ELTE Eötvös Loránd University, Budapest, Hungary; 3 Department of Child and Adolescent Psychiatry, Center of Expertise on Gender Dysphoria, Amsterdam University Medical Centers, Location Vumc, Van der Boechorststraat 7, 1081 BT Amsterdam, The Netherlands; 4 Institute for Mental Health and Wellbeing, College of Education, Psychology and Social Work, Flinders University, Adelaide, South Australia; 5 Centre of Excellence in Responsible Gaming, University of Gibraltar, Gibraltar, Gibraltar; 6 14th District Medical Center, Child and Adolescent Psychiatry, Budapest, Hungary

**Keywords:** Non-suicidal self-injury, Gender minority stressors, Transgender

## Abstract

**Background:**

Transgender and gender diverse (TGD) individuals experience gender-related distal (i.e., discrimination, victimization, rejection, non-affirmation) and proximal (i.e., internalized transphobia, expectation of rejection, and identity nondisclosure) stressors, which increase their risk for non-suicidal self-injury (NSSI). Yet, there is a paucity of research examining the prevalence, characteristics, and correlates of NSSI in TGD populations.

**Methods:**

A convenient sample of Hungarian TGD adults (*N* = 202; *M* = 29.60, SD = 10.27; 39.6% gender diverse individuals, 35.6% trans men, 24.8% trans women) took part in an online, questionnaire-based, cross-sectional survey. Gender minority stressors and several aspects of NSSI (i.e., prevalence, frequency, number of methods, and motivations) were assessed.

**Results:**

Results showed a high lifetime (*n *= 142, 70.3%) and past-month (*n* = 48, 33.8%) prevalence of NSSI, with trans men (*n *= 55, 76.4%) and gender diverse individuals (*n *= 59, 73.8%) reporting higher lifetime prevalence than trans women (*n *= 28, 56.0%). Those who engaged in NSSI previously were significantly younger compared to those without NSSI. Structural equation model (SEM), examining the associations among gender minority stressors and functions of NSSI, revealed that proximal stressors mediated the link between distal stressors and intrapersonal functions of NSSI. Regarding interpersonal functions, neither distal nor proximal stressors were significant predictors.

**Conclusions:**

Findings suggest that trans men and gender diverse individuals, and younger TGD individuals are at greater risk for NSSI. Intrapersonal functions of NSSI and their potential association with proximal stressors should be carefully considered and assessed in clinical practice. Interventions targeting these specific associations should be developed and implemented to provide culturally sensitive care to TGD population.

## Introduction

### Characteristics of non-suicidal self-injury and its associations with gender minority stressors among hungarian trans adults

Non-suicidal self-injury (NSSI) is defined as deliberate, self-inflicted harm to one’s body tissue without suicidal intent [33]. Typical manifestations of NSSI include behaviors such as cutting, burning, scratching, or hitting oneself [9]. Previous research has shown that individuals engage in NSSI for various functions. One of the early models of NSSI functions was the four-function model (FFM), categorizing NSSI motivations in four different categories, which fall along two dichotomous dimensions: negative versus positive and automatic versus social [34]. Although FFM received great attention from scholars [6], the conceptual and psychometric empirical overlap among the four functions was not fully supported. Thus, Klonsky and colleagues [26] proposed a two-function model, categorizing the functions as intrapersonal and interpersonal.

Intrapersonal functions of NSSI refer to ways individuals use self-injury to manage internal emotional or psychological states. These functions are often aimed at regulating emotions, alleviating internal distress, or fulfilling specific psychological needs such as self-punishment, anti-suicide (i.e., using NSSI to cope with suicidal urges or avoid suicidal behavior), and anti-dissociation (i.e., using NSSI to counteract feelings of dissociation; [25]). Interpersonal functions of NSSI, on the other hand, relate to how individuals use self-injury to influence, communicate with, or elicit responses from others in their social environment [26]. These functions often reflect attempts to manage social dynamics, express needs that may be difficult to verbalize or address interpersonal distress. Key interpersonal functions of NSSI include setting boundaries, communicating psychological pain, or bonding with peers [25].

While NSSI is observed in many populations, it is disproportionately common among transgender and gender diverse (TGD) individuals. TGD is an umbrella term that is used to define individuals whose gender identity doesn’t align with their birth-assigned sex [44]. The term includes binary trans identities (i.e., trans men and trans women) as well as identities outside gender binary such as non-binary, genderqueer, and agender. A systematic review [29] found high rates of NSSI among TGD individuals, with lifetime prevalence rates often cited as 60% to 80%. Similarly, another study [10] reported that around 60% of TGD participants had engaged in NSSI. Consistent with these figures, a recent study using a US probability sample found that 50% of TGD participants reported engaging in NSSI [20]. Across various cisgender samples, on the other hand, lifetime NSSI prevalence was estimated to be around 13% to 23% in adolescents and young adults and 5% to 10% in adults [41], suggesting that NSSI prevalence among TGD individuals is significantly higher than in cisgender populations.

However, the prevalence rates and characteristics of NSSI within TGD subgroups—such as trans men, trans women, and gender diverse individuals—have been less frequently studied in detail. Evidence suggests that trans men and gender diverse individuals, in particular, may experience higher rates of NSSI compared to trans women [29]. In one study [10], nearly 60% of TGD individuals reported engaging in NSSI at some point in their lives, with trans men and gender diverse individuals reporting the highest lifetime prevalence rates. This disparity suggests that certain subgroups within the TGD population may be particularly vulnerable to engaging in NSSI.

One major framework for understanding the heightened rates of NSSI in TGD populations is the gender minority stress model, which posits that TGD individuals face unique, socially-based, and chronic stressors that increase their risk for mental health difficulties [18]. These stressors are classified into two main types: distal and proximal. Distal stressors are objective, prejudice-based events that occur in the social environment and include discrimination, victimization, rejection, and non-affirmation of gender identity. On the other hand, proximal stressors are subjective experiences of distal stressors that alter the cognitive, emotional, and behavioral processes of the stigmatized. These include internalized transphobia, expectations of rejection, and identity concealment. Together, these minority stressors create a heightened vulnerability to various adverse mental health outcomes, including NSSI.

The relationship between NSSI and gender minority stress is well-documented, with numerous studies indicating that those who reported more frequent exposure to victimization [10] and transphobic discrimination [4], high levels of felt or anticipated rejection [22], and higher identity concealment and internalized transphobia [21, 31] were significantly more likely to engage in NSSI. While these studies have significantly advanced our understanding of the relationship between gender minority stress and NSSI, how diverse types of minority stressors influence the reasons why TGD individuals engage in NSSI—whether for intrapersonal or interpersonal purposes—is underexplored.

Several studies reported that distal stressors such as discrimination, victimization, and rejection contribute to internal emotional distress and negative self-perceptions, which drive the use of NSSI for more intrapersonal functions such as emotion regulation and self-punishment [23, 40]. Some other studies [10, 12], on the other hand, suggest that distal stressors are more associated with interpersonal functions, since NSSI acts as a way to nonverbally react to prejudice-based events, signal a need for respect, or to make emotional needs visible to others, particularly in settings where individuals’ identity is routinely denied or invalidated.

Regarding proximal stressors, previous evidence suggests a stronger association with intrapersonal functions than interpersonal ones. For instance, higher levels of internalized transphobia and identity concealment were associated with the emotion regulation function of NSSI [31], suggesting that using NSSI as a means of affect regulation could provide a temporary release or distraction from chronic self-directed feelings of transphobia and anxiety and distress around concealing one’s identity. 

Despite this theoretical understanding, there remains a lack of systematic research examining how specific minority stressors, both distal and proximal, relate to different functions and patterns of NSSI within diverse TGD populations. Moreover, most studies examining the relationship between gender minority stress and the functions of NSSI among TGD individuals have been conducted in Western countries, where sociopolitical and cultural contexts may differ significantly from those in other regions [47]. This geographic difference limits the generalizability of findings, particularly in contexts like Hungary, where TGD individuals face unique and substantial legal and social challenges.

In Hungary, the situation for TGD individuals is particularly challenging due to legal and social barriers. In 2020, Hungary passed legislation that makes it illegal for individuals to change their gender marker on official documents (ILGA-Europe the European region of the international lesbian, gay, bisexual, trans and intersex association, [19]. This policy effectively denies legal recognition of trans identities, which can lead to increased stigmatization, discrimination, and exposure to various distal stressors in everyday life [7]. This legal restriction is likely to exacerbate the minority stress experienced by TGD individuals in Hungary, as it limits their ability to live openly and authentically, making it harder for them to access employment, education, and healthcare without facing discrimination [14]. In light of these challenges, there is an urgent need to investigate the experiences of Hungarian TGD individuals, which will address an important gap in the literature and support the development of culturally relevant and sensitive interventions.

A final critical factor to consider is the role of sociodemographic variables, particularly age, in NSSI prevalence among TGD individuals. Younger age has been identified as a risk factor for NSSI in the general population due to the unique developmental challenges of adolescence and young adulthood [32]. Younger TGD individuals are often at even higher risk for engaging in NSSI [10, 12], potentially due to additional exposure to stigma during formative years, challenges with identity acceptance, and lack of supportive resources [11]. Research shows that younger TGD individuals report more frequent experiences of both distal stressors, such as discrimination in school settings [30], and proximal stressors, such as the need to conceal their identity from unsupportive family members [24]. These compounded stressors during critical developmental stages may increase the likelihood of engaging in NSSI as a coping mechanism for managing the complex and often painful process of gender identity development in an unsupportive environment [11].

### Current study

**Fig. 1:**
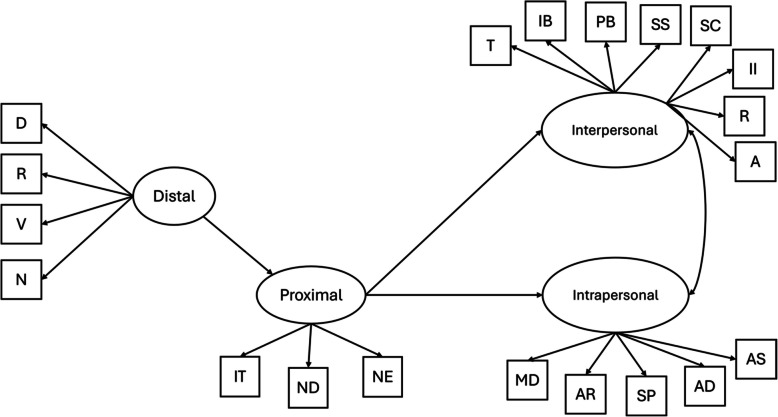
Proposed model of the associations between gender minority stressors and interpersonal functions of NSSI, distal minority stressors as main predictor and proximal minority stressors as mediator. *Note*. D = Discrimination; R = Rejection; V = Victimization; *N* = Non-affirmation; IT = Internalized Transphobia; ND = Non-disclosure; NE = Negative Expectations; T = Toughness; IB = Interpersonal Boundaries; PB = Peer Bonding; SS = Sensation Seeking; SC = Self-care; II = Interpersonal Influence; Rev = Revenge; A = Autonomy; MD = Marking Distress; AR = Affect Regulation; SP = Self-punishment; AD = Anti-dissociation; AS = Anti-suicide

Given the high prevalence of NSSI among TGD individuals, particularly among younger trans men and gender diverse individuals, and the profound role that gender minority stressors appear to play in this, there is an urgent need for research that systematically examines the nuanced associations between gender minority stressors and NSSI. Thus, this study aims to explore the prevalence and characteristics of NSSI within a Hungarian sample of TGD adults and to examine the associations between distal and proximal gender minority stressors and the functions of NSSI. Based on the literature presented above, we hypothesized that 1) lifetime prevalence of NSSI would be more common among trans men and gender diverse individuals than trans women, 2) younger age would be associated with a higher prevalence of NSSI, 3) distal and proximal stressors would predict intrapersonal and interpersonal functions of NSSI, and that 4) proximal stressors would further mediate the association of distal stressors with intrapersonal and interpersonal functions of NSSI. Figure 1 shows the proposed mediation model.

## Method

### Participants and Procedure

The data are from the first data collection point of a longitudinal study. The questionnaire battery was uploaded on Qualtrics and promoted through social media accounts of Hungarian transgender non-governmental organizations, Facebook groups for trans individuals in Hungary, and the website of the LGBTIQ Section of the Hungarian Psychological Association. Eligibility criteria included being at least 18 years old, fluent in Hungarian to complete the survey, and self-identifying as a trans person. During October 2023, 238 Hungarian TGD individuals started to respond to the questionnaire package, 202 of whom completed the survey and were included in the analyses. Participants’ ages ranged from 18 to 74 years (*M* = 29.60, SD = 10.27). Gender diverse individuals represented 39.6% of the sample (*n* = 80), trans men represented 35.6% (*n* = 72), and trans women represented 24.8% (*n* = 50). Participant characteristics are provided in Table 1.

**Table 1:** Sociodemographic characteristics of the sample

	Trans Men (*n* = 72, 35.6%)	Trans Women (*n* = 50, 24.8%)	Gender Diverse Individuals (*n* = 80, 39.6%)
Age, *M (SD)*	27.93 (9.78)	33.70 (11.05)	28.54 (9.62)
Relationship Status, *n (%)*			
Single	42 (58.3)	29 (58.0)	54 (67.5)
In a Relationship	30 (41.7)	21 (42.0)	26 (32.5)
Minority Status, n (%)			
Ethnic Minority	3 (4.2)	3 (6)	3 (3.75)
Religious Minority	6 (8.3)	2 (4)	11 (13.75)
Minority in terms of Disability	10 (13.8)	0 (0)	15 (18.75)
Other Minority	3 (4.2)	2 (4)	8 (10)
Place of Residence, *n (%)*			
Urban	47 (65.3)	35 (70.0)	61 (76.3)
Rural	25 (34.7)	15 (30.0)	19 (23.8)
Education Level, *n (%)*			
Highschool or lower	45 (62.5)	24 (48.0)	51 (63.7)
University degree or higher	27 (37.5)	26 (52.0)	29 (36.3)
Employment Status, *n (%)*			
Employed	46 (63.9)	38 (76.0)	52 (65.0)
Unemployed	26 (36.1)	12 (24.0)	28 (35.0)
Legal Gender Change, *n (%)*			
Yes	12 (16.7)	11 (22.0)	5 (6.2)
No	60 (83.3)	39 (78.0)	75 (93.8)
Gender-Affirming Intervention, *n (%)*			
Yes	35 (48.6)	28 (53.8)	16 (19.8)
No, but I want to	26 (36.1)	12 (23.1)	37 (45.7)
No, and I don’t want	11 (15.3)	12 (23.1)	28 (34.6)

Participation in the study was voluntary and anonymous. All of the respondents gave informed consent to participate in the study. The research plan was approved by the ELTE Research Ethics Committee of Eötvös Loránd University, Faculty of Education and Psychology, and the study was carried out in accordance with the Declaration of Helsinki [50].

### Measures

#### Gender identity

Participants reported their current gender identity using the following response options: “woman/feminine,” “man/masculine,” “non-binary,” and “other.” They also indicated whether they identified as a transgender person, selecting from: “yes, I am a trans woman,” “yes, I am a trans man,” “yes, I am a non-binary trans person,” “yes, I am a trans person with the following identity,” or “no, I am not a trans person.” Following the approach used in prior research (e.g., [17, 43]), participants were categorized into three groups—trans women, trans men, and gender diverse—based on their responses to gender identity and trans status questions.

#### Demographic information

Participants provided sociodemographic and gender identity-related information through a sociodemographic form prepared by the authors. The first section included questions on age, place of residence, relationship status, education level, employment status, and ethnicity, religion, disability, and other minority statuses. The second section gathered information on legal gender change, name change, and gender-affirming intervention status.

### Gender minority stress

Self-reported distal and proximal stressors among TGD individuals were measured by the Hungarian version of the 58-item Gender Minority Stress and Resilience Measure [43]. Four subscales comprise distal stressors: Gender-related discrimination (D; e.g., I have experienced difficulty getting identity documents that match my gender identity), Gender-related rejection (R; e.g., I have been rejected or distanced from friends because of my gender identity or expression), Gender-related victimization (V; e.g., I have had my personal property damaged because of my gender identity or expression), and Non-affirmation of gender identity (NA; e.g., I have difficulty being perceived as my gender). Three subscales refer to proximal stressors: Internalized transphobia (IT; e.g., I resent my gender identity or expression), Negative expectations for future events (NE; e.g., If I express my gender identity, most people would look down on me), and Non-disclosure (ND; e.g., Because I don't want others to know my gender identity, I modify my way of speaking). For D, R, and V subscales, response options contain “*yes, before age 18*”, “*yes, after age 18*”, “*yes, in the past year*”, and “*never*”. Responses were coded as 0 if “never” and as 1 if “yes” at any point is selected. For the other subscales, the responses are on a 5-point scale with options ranging from 0 (*strongly disagree*) to 4 (*strongly agree*). In past research, reliability scores for subscales ranged from adequate to excellent (e.g., [1, 38], similarly in this study (i.e., Cronbach’s alpha values ranged from .62 to .90). 

### Non-suicidal self-injury

The Hungarian version of the Inventory of Statements About Self-Injury [25, 36] was used to get a comprehensive assessment of NSSI. The first section of the two-part questionnaire can measure the lifetime frequency of 12 NSSI methods (e.g., cutting, biting, hitting self) and several additional aspects of NSSI (e.g., whether someone wanted to stop engaging in NSSI or experienced pain during NSSI). The second section of the ISAS outlines 13 different motivations of NSSI, grouped into two broad (i.e., intrapersonal and interpersonal) functional areas. We used in this study the short version of the ISAS Part II [48]. All motivations are assessed by two items on a 3-point scale (0 = *not relevant*, 1 = *somewhat relevant*, 2 = *very relevant*). Good reliability coefficients were determined for both factors in former studies (α_intrapersonal_ = .80; α_interpersonal_ = .87; [15]). In this study, Cronbach alphas were similarly acceptable: α = .79 for the intrapersonal, and α = .77 for the interpersonal factor. In a supplementary question, frequency of NSSI (i.e., number of days on which NSSI act(s) occurred) during the past month was assessed. 

### Data analysis

Descriptive statistics, group comparisons with independent sample t-test and Chi-square test, and the bivariate Pearson’s correlation coefficients between the study variables were calculated by SPSS version 29. Structural equation modeling (SEM) was used for model testing by MPlus version 8. . Maximum likelihood with robust standard errors (MLR) was used for model testing. Following previous similar research [46], we included theoretically relevant sociodemographic variables as control variables, including education level, relationship status, and place of residence. 

For the whole sample, the amount of missing data ranged from 0% (e.g., age, education level etc.) to 1.98% (e.g. nondisclosure, negative expectations). For NSSI subsample (i.e., those who engaged in NSSI at least once in their lifetime, *n* = 142), the amount of missing data ranged from 0% (e.g., age of onset) to 4.41% (e.g., intrapersonal functions, interpersonal functions). Missing values were treated with full information maximum likelihood.

Indirect effects were considered to be significant if the 95% confidence intervals (CI) did not include zero. We followed previous general recommendations to assess the goodness of fit of each model, which were chi-square/degrees of freedom (χ2/df), root mean square error of approximation (RMSEA; < 0.05), standardized root mean square residual (SRMR; < 0.10), comparative fit index (CFI; > 0.95 or > 0.90), and Tucker-Lewis index (TLI; > 0.95 or > 0.90).

## Results

### Descriptive statistics of the study variables

Descriptive statistics and bivariate Pearson’s correlation coefficients between the study variables are presented in Table 2. All variables are found to be relatively normally distributed and Pearson’s correlation coefficients were significant in the expected directions. The only exception to this was the interpersonal functions of NSSI subscale, which was positively correlated with only intrapersonal functions subscale. The correlations between GMSR subscales and interpersonal functions were not significant.

**Table 2:** Descriptive statistics and correlations between the study variables

Variable	1	2	3	4	5	6	7	8	9	*M (SD)*	Skew	Kurt
1. Discrimination	–									2.27 (1.61)	.18	–1.05
2. Rejection	.54^**^	–								2.64 (1.65)	–.06	–.86
3. Victimization	.44^**^	.45^**^	–							1.91 (1.56)	.63	–.47
4. Nonaffirmation	.29^**^	.24^**^	.12	–						13.64 (7.17)	–.42	–.98
5. IT	.17^*^	.29^**^	.24^**^	.39^**^	–					14.41 (7.98)	.19	–.74
6. NE	.33^**^	.31^**^	.28^**^	.42^**^	.46^**^	–				18.84 (8.32)	–.18	–.55
7. Nondisclosure	.42^**^	.38^**^	.23^**^	.23^**^	.39^**^	.49^**^	–			9.47 (5.74)	.04	–.95
8. Intrapersonal	.15	.22*	.11	.26^**^	.34^**^	.17^*^	.37^**^	–		19.68 (4.51)	–.10	–.62
9. Interpersonal	.03	.15	.11	.05	.09	.07	.11	.43^**^	–	20.07 (4.03)	1.29	1.263

*IT* Internalized Transphobia, *NE* Negative Expectations for Future, *Skew*. Skewness, *Kurt*. Kurtosis. *p < .05, **p < .01

## Descriptive statistics of NSSI behaviors

A high proportion of Hungarian TGD respondents, 70.3% (*n* = 142), reported that they had engaged in NSSI at least once in their lifetime. Only 29.7% (*n* = 60) indicated that they had no history of NSSI. One-third (33.8%; *n* = 48) with NSSI history have engaged in NSSI within the past month (i.e., current NSSI behavior), while 66.2% (*n* = 94) engaged in NSSI previously. Most of the current NSSI sample (62,5%, *n* = 30) did so from 1 to 5 days in the past month, another 16.7% (*n* = 8) reported NSSI between 6 and 10 days, 6.3% (*n* = 3) between 11 and 15 days, while 14.5% (*n* = 7) more than 15 days (Table 3).

**Table 3:** Descriptive statistics of NSSI behaviors

	Total (*N* = 202)	Trans Men (*n* = 72, 35.6%)	Trans Women (*n* = 50, 24.8%)	Gender Diverse Individuals (*n* = 80, 39.6%)
Lifetime prevalence *n (%)*	142 (70.3)	55 (76.4)	28 (56.0)	59 (73.8)
Last-month prevalence *n (%)*	48 (33.8)	20 (36.4)	7 (25.0)	21 (35.6)
Last NSSI *n (%)*				
1–5 days	30 (21.1)	11 (20.0)	4 (14.3)	15 (25.4)
6–10 days	8 (5.6)	3 (5.5)	0 (0)	5 (8.5)
11–15 days	3 (2.1)	2 (3.6)	1 (3.6)	0 (0)
15 + days	7 (4.9)	4 (7.3)	2 (7.1)	1 (1.7)
Age of onset *M (SD)*	13.54 (5.08)	12.63 (4.30)	15.48 (7.18)	13.52 (4.38)
Methods *n (%)*				
Hitting self	101 (71.1)	46 (83.6)	13 (46.4)	42 (71.2)
Biting	82 (57.7)	30 (54.5)	15 (53.6)	37 (62.7)
Cutting	81 (57.0)	42 (76.4)	11 (39.3)	28 (47.5)
Burning	45 (31.7)	20 (36.4)	8 (28.6)	17 (28.8)
Carving	59 (41.5)	26 (47.3)	10 (35.7)	23 (39.0)
Pinching	71 (50.0)	24 (43.6)	13 (46.4)	34 (57.6)
Pulling hair	34 (23.9)	17 (30.9)	3 (10.7)	14 (23.7)
Severe scratching	77 (54.2)	30 (54.5)	12 (42.9)	35 (59.3)
Interfering with wound healing	75 (52.8)	31 (56.4)	12 (42.9)	32 (54.2)
Rubbing skin against rough surface	33 (23.2)	13 (23.6)	5 (17.9)	15 (25.4)
Sticking self with needles	31 (21.8)	12 (21.8)	6 (21.4)	13 (22.0)
Swallowing dangerous substances	12 (8.5)	4 (7.3)	4 (14.3)	4 (6.8)
Number of methods *M (SD)*	4.95 (2.71)	5.38 (2.89)	4.04 (2.53)	4.98 (2.55)
Repetitive NSSI *n (%)*	128 (90.1)	48 (87.3)	26 (92.9)	54 (91.5)
Experienced pain *n (%)*				
Always	80 (58.8)	29 (53.7)	16 (61.5)	35 (62.5)
Occasionally	47 (34.6)	23 (46.2)	8 (30.8)	16 (28.6)
Never	9 (6.6)	2 (3.7)	2 (7.7)	5 (8.9)
Being alone *n (%)*				
Always	110 (80.9)	44 (81.5)	21 (80.8)	45 (80.4)
Sometimes	24 (17.6)	9 (16.7)	4 (15.4)	11 (19.6)
Never	2 (1.5)	1 (1.9)	1 (3.8)	0 (0)
Delaying urge *n (%)*				
Less than one hour	75 (55.1)	30 (55.6)	13 (50.0)	32 (57.1)
More than a day	28 (20.6)	7 (13.0)	8 (30.8)	13 (23.2)
Willingness to stop *n (%)*				
Yes	114 (83.8)	47 (87.0)	21 (80.8)	46 (82.1)
No	22 (16.0)	7 (13.0)	5 (19.2)	10 (17.9)
Functions *M (SD)*				
Emotion regulation	5.04 (1.29)	5.09 (1.44)	4.92 (1.26)	5.04 (1.14)
Self-punishment	4.26 (1.48)	4.50 (1.51)	3.92 (1.35)	4.20 (1.49)
Anti-dissociation	3.99 (1.58)	4.09 (1.56)	3.65 (1.52)	4.05 (1.63)
Anti-suicide	3.43 (1.25)	3.41 (1.33)	3.42 (1.27)	3.45 (1.17)
Marking distress	2.96 (1.03)	3.19 (1.03)	2.62 (0.98)	2.89 (1.02)
Peer bonding	2.13 (0.45)	2.17 (0.51)	2.04 (0.20)	2.13 (0.47)
Revenge	2.22 (0.64)	2.13 (0.44)	2.27 (0.83)	2.29 (0.71)
Interpersonal boundaries	2.71 (1.01)	2.93 (1.15)	2.42 (0.76)	2.63 (0.95)
Self-care	2.40 (0.88)	2.37 (0.81)	2.46 (0.99)	2.39 (0.91)
Sensation seeking	2.42 (0.89)	2.37 (0.65)	2.27 (0.83)	2.54 (1.10)
Interpersonal influence	2.86 (1.22)	2.96 (1.35)	2.81 (1.10)	2.79 (1.71)
Toughness	2.96 (1.16)	3.11 (1.18)	2.88 (1.14)	2.86 (1.17)
Autonomy	2.38 (0.84)	2.37 (0.85)	2.23 (0.59)	2.45 (0.93)

There was a significant difference between trans men, gender diverse individuals, and trans women in the lifetime prevalence of NSSI (χ^2^(2) = 6.63, *p* = 0.036, φ = 0.18). Confirming our first hypothesis, we found that trans men (76.4%; *n* = 55) and gender diverse individuals (73.8%; *n* = 59) tended to engage in NSSI at a higher rate than trans women throughout their lives (56.0%; *n* = 28). On the contrary, there was no significant difference between the three groups in the rate of past month (i.e., current) prevalence of NSSI (χ^2^(2) = 1.22, *p* = 0.545, φ = 0.09; 36.4% of trans men (*n* = 20); 35.6% of gender diverse individuals (*n* = 21) and 25.0% of trans women (*n* = 7)).

The average age when TGD respondents engaged in NSSI at the first time was 13.54 years (*SD* = 5.08), with the highest prevalence between the ages of 10 and 16. Furthermore, confirming our second hypothesis, we found that those who ever engaged in NSSI (*M*_age_ = 27.11, *SD* = 8.52) were significantly younger (*t*(83.26) = −5.24, *SE* = 1.68, *p* < 0.001) compared to those without any NSSI history (*M*_age_ = 35.93, *SD* = 11.60). Among those who engaged in NSSI, the most common types of NSSI methods were banging or hitting self (71.1%; *n* = 101), biting (57.7%; *n* = 82) and cutting (57.0%; *n* = 81). Swallowing dangerous substances was the less prevalent method (8.5%; *n* = 12). There were significant differences between TGD groups in the prevalence of two NSSI methods. More trans men (76.4%; *n* = 42) engaged in cutting than gender diverse individuals (47.5%; *n* = 28) and trans women (39.3%; *n* = 11; χ^2^(2) = 14.19, *p* < 0.001, φ = 0.32). While more trans men (83.6%; *n* = 46) and gender diverse individuals (71.2%; *n* = 42) engaged in hitting self than trans women (46.4%; *n* = 13; χ^2^(2) = 12.51, *p* = 0.002, φ = 0.30).

Those who engaged in NSSI in their lifetime applied five NSSI methods on average (*M *= 4.95; *SD *= 2.71; ranged between 1–11 methods). The vast majority (90.1%; *n* = 128) of the self-injurious sample engaged in repetitive NSSI (≥10 lifetime episodes; [16]).

Over half of the sample who engaged in NSSI (58.8%; *n* = 80) experienced pain during self-injury, 34.6% (*n* = 47) experienced pain occasionally, the rest (6.6%; *n* = 9) reported no pain. Most of them were alone during the NSSI episodes (80.9%; *n* = 110), 17.6% (*n* = 24) reported they were sometimes alone, only 1.5% (*n* = 2) indicated they were not alone at that time. About half of the sample (55.1%; *n* = 75) engaged in NSSI in less than an hour when they felt the urge to engaging in NSSI, while 20.6% (*n* = 28) were able to delay more than a day. Most of the TGD individuals who engaged in NSSI would like to stop engaging in these acts (83.8%; *n* = 114), while 15.5% (*n* = 22) thought they never wanted to stop NSSI behaviors.

The most prominent motivations behind NSSI were emotion regulation (*M* = 5.04; *SD* = 1.29), self-punishment (*M* = 4.26; *SD* = 1.48) and generating emotions (e.g., to eliminate dissociative states; *M* = 3.99; *SD* = 1.58). The least relevant functions of NSSI were peer bonding (*M* = 2.12; *SD* = 0.45) and revenge (*M* = 2.22; *SD* = 0.64). There was no significant difference between the three TGD groups in the mean of any NSSI motivations.

### The association of gender minority stressors with NSSI

The first SEM model testing the direct path from distal stressors to intrapersonal and interpersonal functions of NSSI had an acceptable fit: χ^2^(191) = 378.14, *p* < 0.001; RMSEA = 0.07, 90%CI [0.06 – 0.08], SRMR = 0.07, CFI = 0.94, TLI = 0.93. Partially confirming our third hypothesis, distal stressors significantly and positively predicted intrapersonal functions of NSSI (β = 0.14, *p* < 0.05). However, the association with interpersonal functions was insignificant (β = 0.04, *p* = 0.36).

**Fig. 2:**
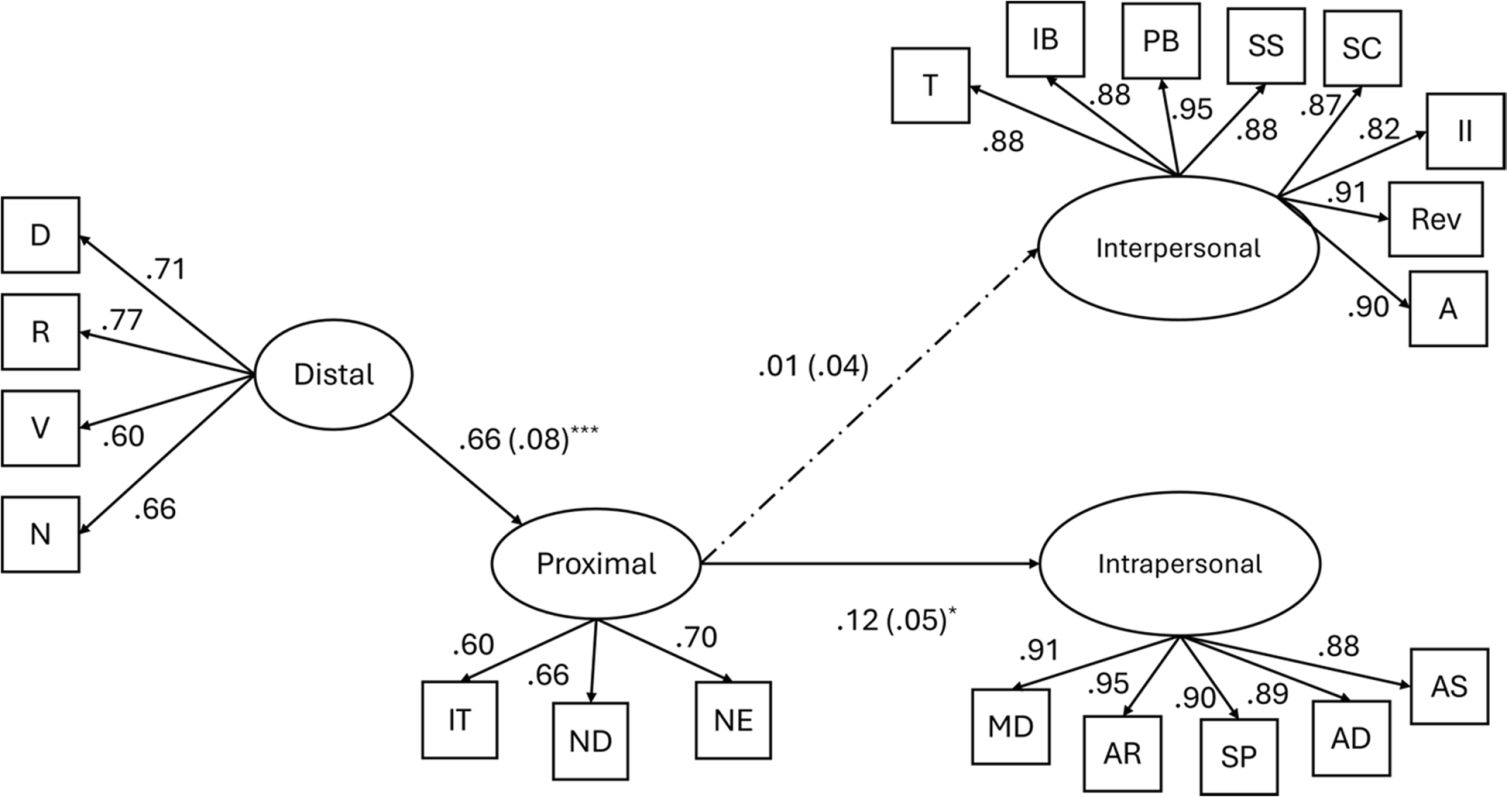
Results from structural equation modeling examining indirect effects of distal stressors on interpersonal and intrapersonal motives of NSSI through proximal stressors. *Note*. D = Discrimination; R = Rejection; V = Victimization; *N* = Non-affirmation; IT = Internalized Transphobia; ND = Non-disclosure; NE = Negative Expectations; T = Toughness; IB = Interpersonal Boundaries; PB = Peer Bonding; SS = Sensation Seeking; SC = Self-care; II = Interpersonal Influence; Rev = Revenge; A = Autonomy; MD = Marking Distress; AR = Affect Regulation; SP = Self-punishment; AD = Anti-dissociation; AS = Anti-suicide. Standardized regression coefficients are reported. Error terms and correlation between error terms are excluded for coherence. Dashed line indicates a nonsignificant relationship. ^***^ *p* < .001, ^*^ *p* < .05

In the second model, when proximal stressors are included as the mediator, the model fit improved: χ^2^(247) = 434.24, *p* < 0.001; RMSEA = 0.06, 90% CI [0.05 – 0.07]; SRMR = 0.06, CFI = 0.95, TLI = 0.94. Partially confirming our hypothesis, proximal stressors significantly mediated (β = 0.08, *p* < 0.05, 95% CI [0.006 – 0.158]) the link between distal stressors and intrapersonal functions of NSSI (see Fig. 2). In other words, distal stressors significantly and positively predicted proximal stressors (β = 0.66, *p* < 0.001), which in turn was associated with higher levels of intrapersonal functions of NSSI (β = 0.12, *p* < 0.05). After including proximal stressors in the model, the direct effect of distal stressors on intrapersonal functions of NSSI became nonsignificant (β = –0.01, *p* = 0.92). However, proximal stressors did not significantly mediate the association of distal stressors with interpersonal functions of NSSI (β = 0.01, *p* = 0.81, 95% CI [–0.047—0.060]). Including covariates did not change the overall pattern of results. Thus, they were excluded from the final model for the principle of parsimony.

## Discussion

The present study aimed to examine the prevalence and characteristics of NSSI and its associations with gender minority stress among TGD individuals in Hungary. The findings contribute to the growing literature on the mental health challenges faced by TGD populations, highlighting significant gaps in understanding the nuanced relationships between gender minority stressors and NSSI.

First, our results indicate a high prevalence of NSSI among TGD individuals, consistent with prior research conducted in other Western contexts [10, 13]. Lifetime engagement in NSSI was reported by 70.3% of the sample, with particularly high rates among trans men (76%) and gender diverse individuals (74%) compared to trans women (56%). Although these numbers reflect gender differences reported in previous research, the reasons behind these gender differences are not fully understood. One hypothesis is that biological factors associated with being phenotypically female may influence emotion dysregulation [29], as NSSI is more common in cisgender women than men [10]. Yet, future studies incorporating biopsychosocial factors are needed to shed light on these gender differences. 

Last month prevalence of NSSI was also high, reported by 33.8% of the individuals who have ever engaged in NSSI. These figures exceed those reported for cisgender populations [41] and are comparable to previously reported prevalence rates in TGD populations [20, 29], underscoring the unique vulnerabilities of TGD individuals. Notably, as expected, younger participants were more likely to engage in NSSI, reflecting trends observed in broader age-related studies of NSSI [12]. These findings suggest a critical need for early intervention and age-appropriate mental health resources tailored to transgender youth.

The study’s findings are in line with the theoretical framework of gender minority stress [18], revealing that both distal and proximal stressors are associated with NSSI [2, 37]. Proximal stressors were particularly significant in mediating the relationship between distal stressors and intrapersonal functions of NSSI, such as emotion regulation and self-punishment. This aligns with previous findings that emphasize the role of internalized transphobia [31] and emotion dysregulation [8] in NSSI among TGD individuals. 

Interestingly, while distal and proximal stressors were linked to intrapersonal functions, their connection to interpersonal functions of NSSI (e.g., setting boundaries or seeking social support) was less pronounced. This distinction underscores the importance of addressing specific stressors and their functional NSSI outcomes in therapeutic settings. However, the lack of a robust association between gender minority stressors and interpersonal NSSI functions could also be due to a relatively lower prevalence of interpersonal functions in our sample. Nonsignificant correlation coefficients obtained between interpersonal functions and gender minority stressors further supports this argument. Still, it is important to note that this result also reflects previous findings both from the general population [42] and TGD population [28] regarding the prevalence of different functions of NSSI, reporting that interpersonal functions are less common than intrapersonal ones.

This study is among the first to explore the relationships between gender minority stress and NSSI in a Hungarian TGD sample. The sociopolitical climate in Hungary, characterized by pervasive anti-transgender rhetoric and restrictive policies, may exacerbate minority stress and its mental health consequences [47]. Our findings also suggest that systemic discrimination and lack of legal recognition likely amplify distal stressors, which, in turn, contribute to proximal stress experiences, which further are associated with engaging in NSSI for intrapersonal motives. Given the predominance of studies conducted in Western countries, this research highlights the necessity of examining NSSI within diverse cultural contexts. The nonsignificant results regarding interpersonal functions of NSSI might also be related to cultural differences between Hungary and previous Western samples. Thus, future studies should explore how sociopolitical environments interact with individual factors to influence NSSI behaviors and motives.

The findings of this study have important implications for clinical practice. First, they demonstrate the need for culturally sensitive mental health interventions that address both distal and proximal stressors in TGD populations. For this purpose, clinicians should be trained to recognize and address the impact of diverse gender minority stressors, more specifically, internalized transphobia, expectations of rejection, and identity nondisclosure on mental health. Considering the significant mediator role of proximal stressors on the link between distal stressors and intrapersonal functions, proximal stressors appear to be an important contributor to NSSI engagement motives, making them a target for interventions. Thus, specific interventions aimed at reducing proximal stressors, such as cognitive-behavioral techniques to challenge internalized negative beliefs [5], may be particularly effective in mitigating the intrapersonal functions of NSSI.

Additionally, therapeutic approaches should incorporate an understanding of the specific functions of NSSI, with particular emphasis on addressing intrapersonal motivations such as emotion regulation and self-punishment. Given that the higher frequency of intrapersonal motivations mimics the trend observed in the general population, interventions developed specifically for emotion regulation and self-punishment can be incorporated into clinical practice with TGD individuals. For instance, clinicians could provide TGD individuals with adaptive emotion regulation mechanisms [3] and foster resilience to help reduce reliance on NSSI as a maladaptive coping strategy [49].

Several empirically supported therapeutic approaches may be relevant for addressing intrapersonal motivations of NSSI in TGD individuals. For instance, dialectical behavior therapy (DBT) has been widely used for individuals with emotion dysregulation and NSSI behaviors, emphasizing distress tolerance and emotion regulation skills [27]. Previous literature identified several techniques of DBT that might be potentially helpful in clinical practice with TGD individuals, such as the adaptation of body awareness exercises and physiologically-related coping techniques and the reinforcement of self-care skills [45]. Furthermore, clinicians can benefit from the model proposed by Skerven and colleagues [39] that incorporates and adapts traditional DBT techniques to challenge structural, enacted, felt, and internalized stigma among sexual and gender minority populations.

Moreover, because we found high prevalence of NSSI among younger TGD participants, and a relatively early mean age of onset (*M* = 13.54) with the most frequent ages of onset being between 10 and 16, early intervention strategies seem to be essential. Schools, universities, healthcare providers, and community organizations should collaborate to provide affirming and supportive environments for younger TGD individuals [35], which will decrease the minority stressors experienced by this population.

Finally, systemic interventions are needed to address the broader sociopolitical factors contributing to minority stress in Hungary. Advocacy efforts should focus on promoting legal and policy changes to protect trans rights and improve access to affirming healthcare and mental health services [47]. By addressing these systemic barriers, it may be possible to alleviate some of the distal stressors driving proximal stress and, ultimately, reduce the prevalence of NSSI in TGD population.

### Limitations

While this study provides valuable insights, several limitations must be acknowledged. The reliance on self-reported data may introduce recall or social desirability bias, and the cross-sectional design limits our ability to draw causal inferences. Additionally, the sample may not fully capture the experiences of TGD individuals in rural or less accessible areas of Hungary, where minority stress may manifest differently. Similarly, although online data collection increases anonymity and thus willingness to participate, TGD individuals with limited access to the internet could not be reached. Longitudinal studies with more diverse data collection methods are needed to decrease sampling biases, clarify the temporal relationships between minority stress and NSSI functions, and to identify protective factors that may buffer against these stressors.

In a similar manner, although we recruited one of the biggest samples of Hungarian TGD adults, our results were not representative of the population, making results less generalizable. Future studies with more representative samples could increase generalizability and provide a more detailed understanding of intersectional stigma experiences of TGD individuals.

Moreover, although we found worthwhile associations between the study variables, regarding interpersonal functions of NSSI, the association with gender minority stressors were not significant. Thus, future studies utilizing a qualitative approach should be conducted to understand the nuanced associations between gender minority stressors and interpersonal functions of NSSI. Similarly, we found that including proximal stressors in the model changed the effect of distal stressors to nonsignificant, meaning that the way individuals appraise the stressors, rather than the stressors themselves, might be more relevant in the context of NSSI. Thus, further studies including variables that could potentially explain this mechanism, such as emotional sensitivity or cognitive appraisal, might provide a more detailed understanding of gender minority stress–NSSI link.

Although our study provided a detailed picture of NSSI prevalence, characteristics, and motives in a TGD population, most of the results were not comparable to previous findings due to variability in utilized NSSI measurement tools. Thus, future studies with more consistent measurements of NSSI are needed to be able to compare and contrast the complex nature of NSSI behaviors in TGD populations. Also, our study only focused on gender minority stressors, disregarding the importance of protective and resilience factors. Thus, future research should explore the role of social support, community connectedness, and other resilience factors in mitigating the impact of minority stress on NSSI.

Similarly, although we could speculate about the impact of cultural differences, we were not fully able to test these assumptions. Therefore, comparative studies across different cultural and geopolitical contexts are needed to further elucidate how systemic factors influence NSSI among TGD individuals. Finally, the intervention programs mentioned here need to be evaluated in further intervention studies to test the efficacy of these targeted therapeutic approaches in reducing NSSI and overall improving mental health outcomes in the TGD population.

## Conclusion

This study highlights the pervasive impact of gender minority stress on NSSI among TGD individuals in Hungary, emphasizing the urgent need for targeted interventions and policy changes to support this population. By addressing the specific stressors, functional motivations underlying NSSI, and the complex associations between them, clinicians, and policymakers can work towards fostering a more inclusive and supportive environment for TGD individuals to improve their mental health.

## Data Availability

The dataset used and analyzed during the current study is available from the corresponding author on reasonable request.

## Abbreviations

CFI: Comparative Fit Index
CI: Confidence Interval
FFM: Four-function Model
GMSR: Gender Minority Stress and Resilience Measure
ILGA-Europe: The European region of the international lesbian, gay, bisexual, trans and intersex association
ISAS: Inventory of Statements About Self-Injury
NSSI: Non-suicidal self-injury
RMSEA: Root Mean Squared Error of Approximation
SEM: Structural Equation Modeling
SRMR: Standardized Root Mean Square Residual
TGD: Transgender and gender diverse
TLI: Tucker-Lewis Index
